# Naturally derived bioactive compounds as precision modulators of immune and inflammatory mechanisms in psoriatic conditions

**DOI:** 10.1007/s10787-024-01602-z

**Published:** 2024-11-22

**Authors:** Ada Radu, Delia Mirela Tit, Laura Maria Endres, Andrei-Flavius Radu, Cosmin Mihai Vesa, Simona Gabriela Bungau

**Affiliations:** 1 Doctoral School of Biological and Biomedical Sciences, University of Oradea, 410087 Oradea, Romania; 2 Department of Pharmacy, Faculty of Medicine and Pharmacy, University of Oradea, 410028 Oradea, Romania; 3 Department of Preclinical Disciplines, Faculty of Medicine and Pharmacy, University of Oradea, 410073 Oradea, Romania; 4 Department of Psycho-Neurosciences and Recovery, Faculty of Medicine and Pharmacy, University of Oradea, 410073 Oradea, Romania

**Keywords:** Psoriasis, Natural bioactive compounds, Anti-inflammation, Diet, Autoimmune disease

## Abstract

Psoriasis represents a chronic autoimmune skin condition defined by various clinical forms, including inverse, erythrodermic, pustular, guttate, plaque types. While current therapies, including topical treatments but also systemic through conventional synthetic drugs and biologics, have improved symptom management, no treatment completely cures the disease, and numerous options are linked to considerable adverse effects, including immunosuppression and carcinogenic risks. Therefore, there is growing interest in bioactive compounds from natural sources due to their potential to reduce inflammation and oxidative stress in psoriasis with fewer adverse effects. The present narrative review aimed to address the limitations of current psoriasis therapies by exploring the therapeutic potential of bioactive compounds in the classes of flavonoids, terpenoids, omega-3 fatty acids, and alkaloids assessed through complex experimental models, focusing on their immunomodulatory and anti-inflammatory properties. Recent studies highlight the efficacy of natural bioactive compounds in reducing psoriasis symptoms, either as standalone treatments or in combination with conventional therapies. While these compounds show promise in alleviating psoriasis-related inflammation, further research is needed to optimize their therapeutic use, understand their mechanisms of action, and assess long-term safety. Future studies should focus on clinical trials to establish standardized protocols for incorporating bioactive compounds into psoriasis management and explore their potential role in personalized treatment strategies. Continued research is essential to develop more effective, safer, and affordable therapeutic options for psoriasis patients.

## Introduction

Psoriasis represents a chronic, clinically diverse skin condition that manifests in various forms, such as plaque, guttate, pustular, flexural, and erythrodermic types (Raharja et al. 2021). The global prevalence of psoriasis differs significantly across regions. In Taiwan, the prevalence of this condition is as low as 0%, while Italy reports an incidence of approximately 2.1%. For adults, the rates vary significantly, ranging from 0.4% in certain Asian countries to 8.5% in Norway (Armstrong et al. 2021).

**Fig. 1:**
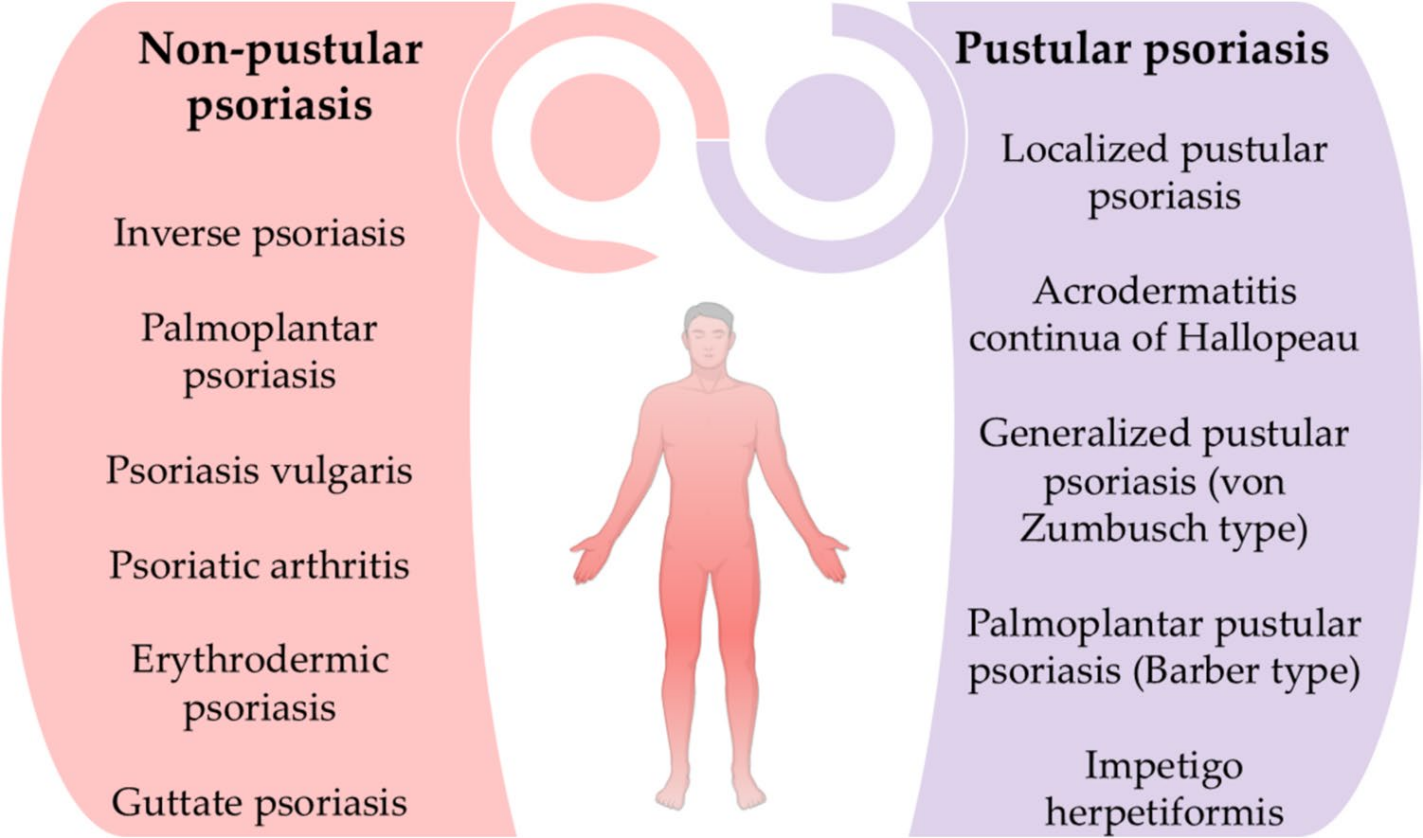
Classification of psoriasis clinical forms

Several distinct types of psoriasis exist, each presenting unique characteristics (National Institute of Arthritis and Musculoskeletal and Skin Diseases 2024). The most prevalent variant of psoriasis is plaque psoriasis, which is defined by the development of raised, red lesions covered with silvery-white scales. These plaques generally manifest symmetrically on the body, often impacting regions such as the scalp, trunk, and extremities, particularly the knees and elbows (Badri et al. 2023). Guttate psoriasis, commonly observed in children and young adults, manifests as tiny, red, teardrop-shaped lesions typically located on the chest and extremities. It is frequently induced by upper respiratory tract infections, especially streptococcal pharyngitis (Zhou et al. 2024). Pustular psoriasis is identifiable by the development of pus-filled blisters encircled by erythematous skin. This variety often impacts the hands and feet, but a more severe variant can disseminate across extensive regions of the body. Pustular psoriasis may be triggered by factors like drugs, infections, stress, or exposure to specific chemicals (Rivera-Díaz et al. 2023). Inverse psoriasis generally manifests as smooth, erythematous lesions in skin folds, such areas beneath the breasts, in the groin, or in the axillae. Friction and perspiration in these regions can intensify symptoms (Micali et al. 2019). Erythrodermic psoriasis is an uncommon although potentially fatal variant, distinguished by extensive erythema and desquamation of the integument. It may be triggered by factors such as intense sunburn or improper use of drugs like corticosteroids. This syndrome frequently arises in persons with inadequately controlled psoriasis (Lo and Tsai 2021). Furthermore, Fig. 1 depicts the clinical classification of psoriasis, targeting two groups: pustular and non-pustular psoriasis (Sarac et al. 2016).

Notable progress in biological therapies has underscored that the interplay of interleukin (IL)-23p19, the IL-17A signaling axis, and tumor necrosis factor-alpha (TNF-α) is fundamental in the context of inflammatory diseases, along with the participation of skin-resident immune cells and essential signaling pathways in the pathogenesis of psoriasis. IL-17-producing T-helper 17 (Th17) cells are not the exclusive contributors; innate lymphoid cells (ILC) type 3 also directly initiate psoriasis rashes. This occurs independently of T-cell participation, as ILC3 reacts to antimicrobial peptides secreted by active keratinocytes and pro-inflammatory cytokines. Nuclear expression of the retinoic acid receptor-related orphan receptor gamma t (RORγt) occurs within these specific cells. Maturation of these cells is stimulated by IL-23 and IL-7, resulting in the production of IL-22 and IL-17 (Bellinato et al. 2021; Yamanaka et al. 2021).

Chronic immunological dysregulation in psoriasis is frequently associated with aberrant antigen presentation, resulting in heightened T-cell activation (Campanati et al. 2021). Comprehending the molecular processes of inflammation in autoimmune disorders is essential for developing successful treatment strategies. The interaction of cytokines in psoriasis can be elucidated by positing a linear correlation among inducers such as IL-23 or IL-12, the synthesis of interferon-gamma and TNF by T-cells, and the ensuing activation of interferon-responsive genes through signal transducer and activator of transcription (STAT) 1 (Wang et al. 2021).

Clinical evaluation is the primary method of diagnosing psoriasis. Among various types of psoriasis, chronic plaque psoriasis stands out as the most common clinical presentation, impacting approximately 80% to 90% of individuals diagnosed with the condition. A variety of other clinical manifestations are also present. Erythematous lesions are the distinguishing feature of classic plaque psoriasis that are symmetrical and finely defined, with a silvery scale. Although they may develop anywhere on the body, these plaques are most frequently observed on the scalp, trunk, buttocks, and extremities. Nail involvement is another prevalent characteristic that may manifest even in the absence of visible cutaneous lesions. Active lesions may occasionally induce discomfort, manifesting as either itching or pain. Psoriasis may also manifest as an isomorphic response, in which new plaques develop on previously unblemished skin because of trauma. The level of severity of psoriasis is an essential consideration for selecting therapeutic approaches, and it can be defined as mild, moderate, or severe (Kim et al. 2017).

Topical treatments remain the cornerstone of psoriasis management, primarily due to their limited side effects. However, biological therapies have revolutionized the treatment landscape, offering greater efficacy and fewer adverse reactions. Additionally, nano-formulations have emerged as a promising alternative, providing enhanced anti-psoriatic effects and fewer side effects compared to conventional therapies. Phyto-pharmaceuticals, which utilize plant-based compounds, are also gaining popularity as complementary and alternative treatments for alleviating psoriasis symptoms (Bakshi et al. 2020).

Despite certain advances, no current treatment fully eradicates psoriasis, and many available therapies are associated with side effects such as skin thinning, organ toxicity, immunosuppression, infection risk, and potential carcinogenesis. This underscores the ongoing need for the development of safer, more effective, and affordable therapeutic approaches (Herman and Herman 2016; Nowak-Perlak et al. 2022). As a result, there is growing interest in herbal therapies, which offer a safer alternative with fewer side effects.

The potential of bioactive substances to modify the immune system's action in psoriasis is a very promising and challenging area of research. Various studies have highlighted the immunoregulatory and antioxidant properties of herbal remedies, supporting their use in managing this autoimmune condition. Literature reviews emphasize the value of phytochemicals in mitigating psoriasis symptoms and their potential role in long-term disease management (Aghmiuni et al. 2017).

In recent years, the use of herbal treatments in more developed regions has grown significantly, demonstrating improved efficacy in managing psoriasis, either as standalone therapies or as adjuncts to synthetic medications. The reduced side effect profile of herbal medicines compared to conventional treatments has contributed to their rising popularity in psoriasis management (Dabholkar et al. 2021).

In the current narrative review, the aim was to deliver a note of update to the current knowledge on the impact of bioactive compounds in managing inflammation in autoimmune conditions like psoriasis. Given the limitations of existing therapies and the fact that psoriasis remains an incurable disease, this paper focuses on the therapeutic potential of natural bioactive compounds as either alternative or complementary treatments. By thoroughly evaluating recent evidence, the review highlights the efficacy and safety of these compounds in reducing inflammation. This work contributes to the scientific literature by providing a fresh perspective on bioactive compounds’ ability to address chronic inflammation in psoriasis, filling a gap in current therapeutic strategies that still have unmet needs.

## Research methodology

**Fig. 2:**
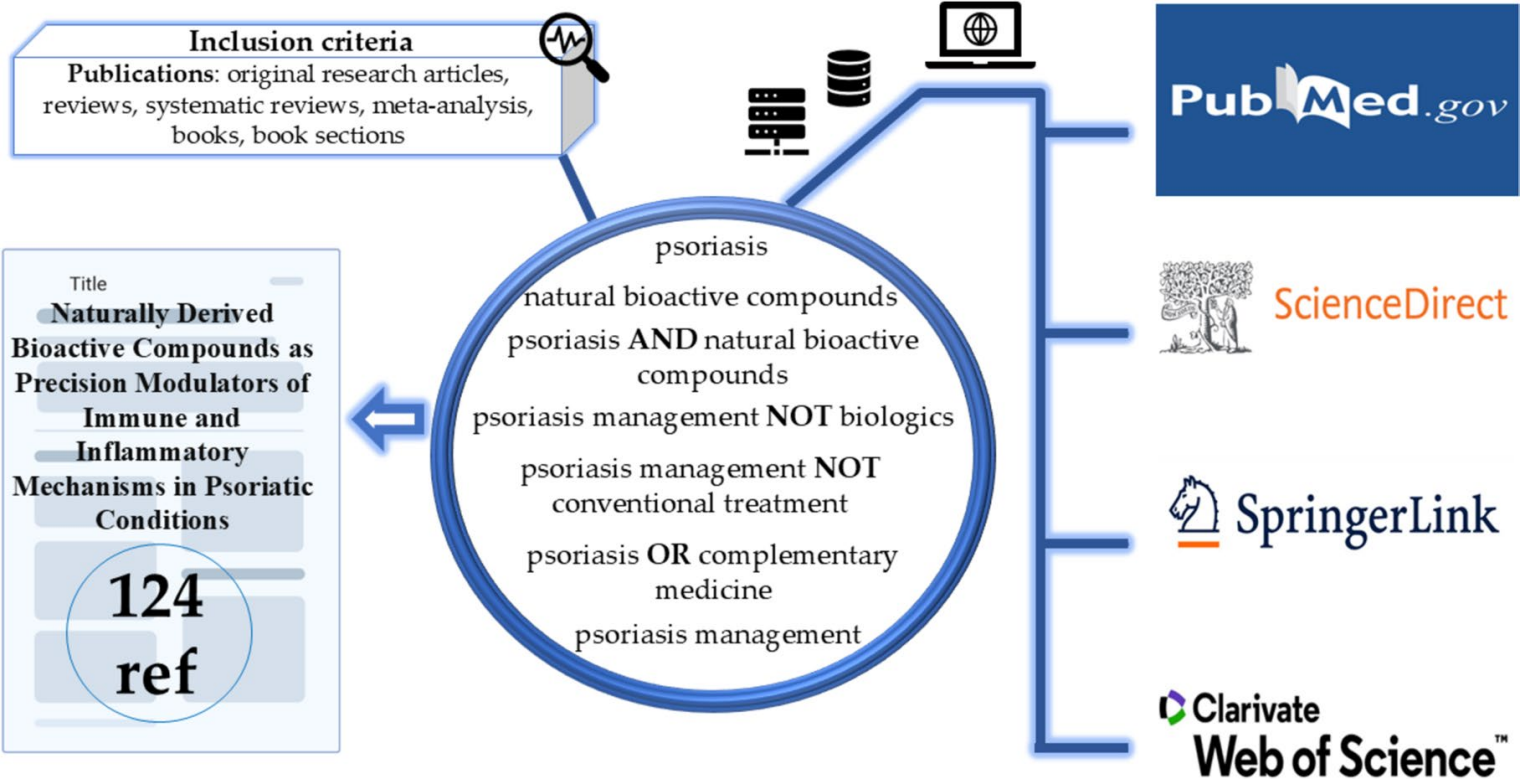
Methodological approaches regarding the selection of the analyzed literature

**Fig. 3:**
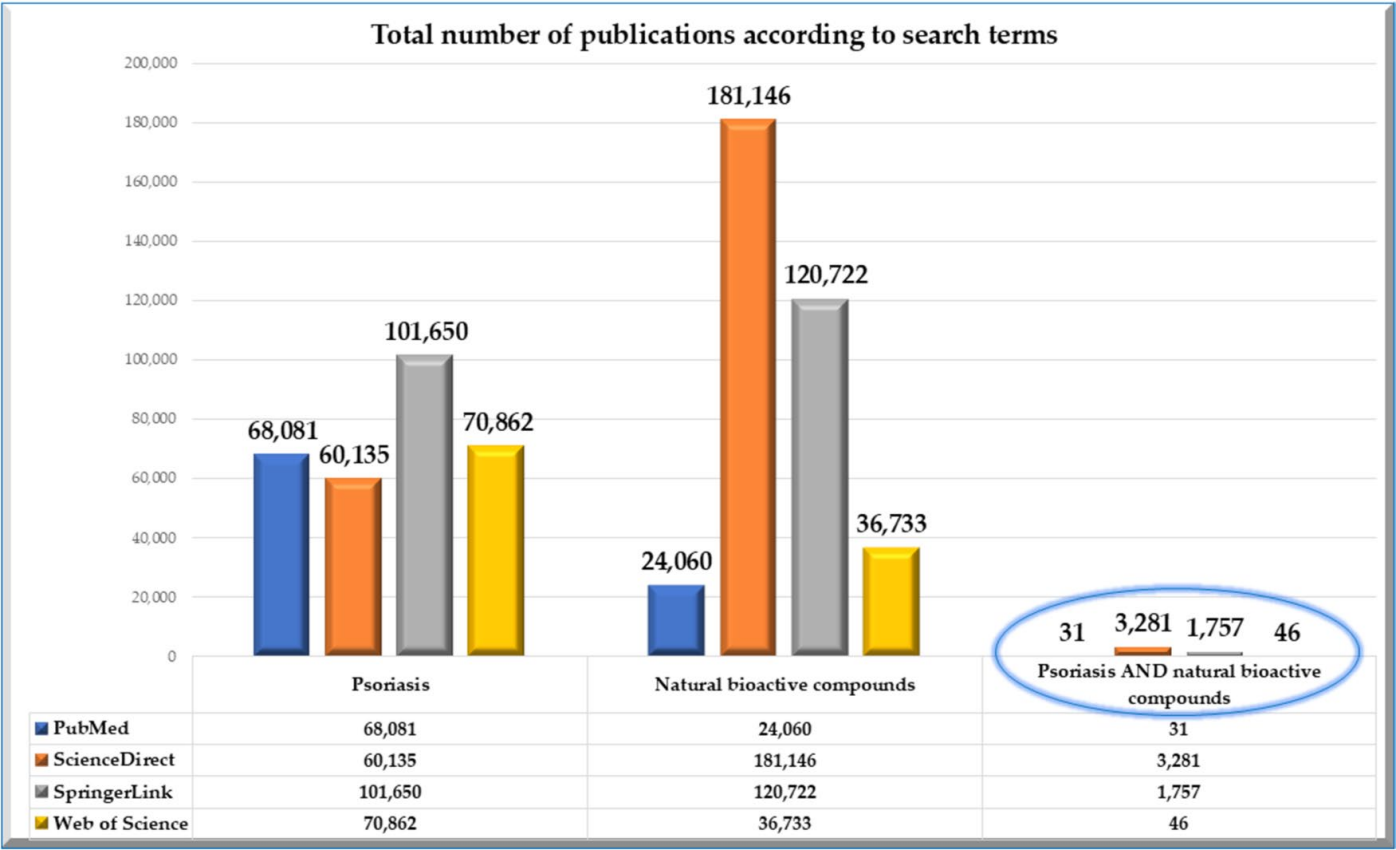
Disparities in publication output across keyword searches highlighting the insufficient focus on addressing psoriasis with naturally derived bioactive substances

The present paper updates the state-of-the-art in the subfield of psoriasis management by natural bioactive compounds with immunomodulatory and anti-inflammatory potential, centralizing and reviewing among the most recent scientific publications in this field. Therefore, we selected scientifically validated databases with high coverage for the topic (i.e., Web of Science, ScienceDirect, SpringerLink, and PubMed) and applied a predefined search algorithm (Fig. 2). The Boolean operators (i.e., AND, OR, NOT) were used to generate the desired search results, to increase the accuracy of the advanced search, and to emphasize that although studies on natural bioactive compounds in general are numerous, the targeting of psoriasis by them is significantly lower, and the continuation and updating of data in this regard becomes essential, especially in the context of the fact that it is an incurable pathology (Fig. 3).

Publications that were not written in English, that were not significantly informative for the purpose of the article, and those that were not included in the category of scientific articles and books were excluded in the literature screening stage. A total of 124 bibliographical references, predominantly from recent years, were selected, analyzed, and included in this review to validate the information presented.

## Pathophysiology of psoriasis and current treatment options

### Pathophysiological mechanisms

Chronic the genetics of complex diseases such as psoriasis have been significantly explored through genome-wide association studies (GWAS). GWAS efficiently genotypes millions of genetic markers across the genome by employing highly refined microarrays. This method is exceedingly sensitive, enabling the identification of even minor variations in allele frequencies between healthy controls and affected individuals, thereby transcending the capabilities of conventional linkage analysis. Thus, the genetic analysis of multifactorial diseases, including psoriasis, has been significantly altered by GWAS (Nair et al. 2009).

Recent improvements have seen the implementation of exome chips to completely analyze protein-altering mutations, including rare ones. Functional single nucleotide polymorphisms (SNPs) were identified within 11 recognized psoriasis susceptibility regions through a meta-analysis of exome chip data from 12,000 psoriasis patients and 29,000 controls. This investigation revealed novel insights into the manner in which rare variants, particularly those located within genes that are involved in type I interferon signaling, such as IFIH1 and TYK2, contribute to the susceptibility to psoriasis (Dand et al. 2017).

The lead SNPs are typically located near genes that influence critical immune pathways, and these susceptibility regions often contain multiple genes. The genes associated with various functions include those related to skin barrier integrity (LCE3B/3D), antigen presentation (HLA-C, ERAP1), and T17 cell activation (TRAF3IP2, IL12B, IL23R, IL23A). Additionally, genes involved in innate antiviral defense and type I interferon signaling, such as RNF114 and IFIH1, are also part of this group (Nair et al. 2009; Strange et al. 2010).

Psoriasis is associated with a complex pathogenesis, driven by multiple immune and genetic factors identified through GWAS. Central to psoriasis development is the dysregulation of the Nuclear Factor kappa-light-chain-enhancer of activated B cells (NF-kB) signaling pathway, a critical regulator of immunological activity. The specific pathway is activated through receptors like Toll-like receptors (TLRs), leading to the recruitment of IKKα, IKKβ, and IKKγ, which phosphorylate IkB proteins, causing their degradation and allowing NF-kB subunits p50 and p65/c-Rel to translocate to the nucleus. Mutations in genes such as REL, TNIP1, TNFAIP3, TRAF3IP2, NFKBIZ, and CARD14 amplify this signaling, contributing to the heightened immune response observed in psoriasis. The Type I interferon signaling pathway, which is often upregulated in psoriasis, further activates NF-kB, intensifying the inflammatory cascade. Another pivotal pathway is the IL-17/IL-23 axis, where genetic variations in IL12B, TNFSF15, IRF4, TYK2, STAT3, IL23R, and IL23A promote activation and even differentiation of Th17 cells. These cells produce IL-17, a cytokine that drives keratinocyte hyperproliferation and inflammation, with significant crosstalk between the IL-17/IL-23 axis and the NF-kB pathway, exacerbating the disease.

Th17 cells are the primary producers of interleukin-17 IL-17, a pro-inflammatory cytokine that is essential for the development of psoriasis. Upon activation, these cells release IL-17A, IL-17F, and other related cytokines, leading to several key actions (Shabgah et al. 2014). IL-17 stimulates keratinocytes to produce a variety of inflammatory mediators, such as IL-6 and antimicrobial peptides (Kuwabara et al. 2017; Mosca et al. 2021). IL-6 acts as a pro-inflammatory cytokine that is upregulated in psoriatic lesions, promoting keratinocyte proliferation and enhancing immune responses, thereby playing a crucial role in sustaining the inflammatory environment in psoriasis (Goodman et al. 2009).

The local immune response is heightened when IL-17 increases the recruitment of neutrophils as well as other cells from the immune system to the area of inflammation. This recruitment is facilitated through the upregulation of chemokines, such as CXCL1 and CXCL8 (Onishi and Gaffen 2010).

IL-17 interacts with the IL-23 signaling pathway, enhancing Th17 cell differentiation and promoting their survival, which intensifies the inflammatory response and sustains the psoriatic condition. In this context, IL-23 is crucial for the initial activation and maintenance of Th17 cells, driving the overall inflammatory process and exacerbating the chronic nature of the disease (Bianchi and Rogge 2019). Moreover, the IL-1 cytokine family, comprising IL-1β, IL-18, IL-33, and IL-36, is integral to psoriasis pathogenesis by facilitating inflammation, Th17 differentiation, and keratinocyte stimulation, thus worsening the condition (Iznardo and Puig 2021).

**Fig. 4:**
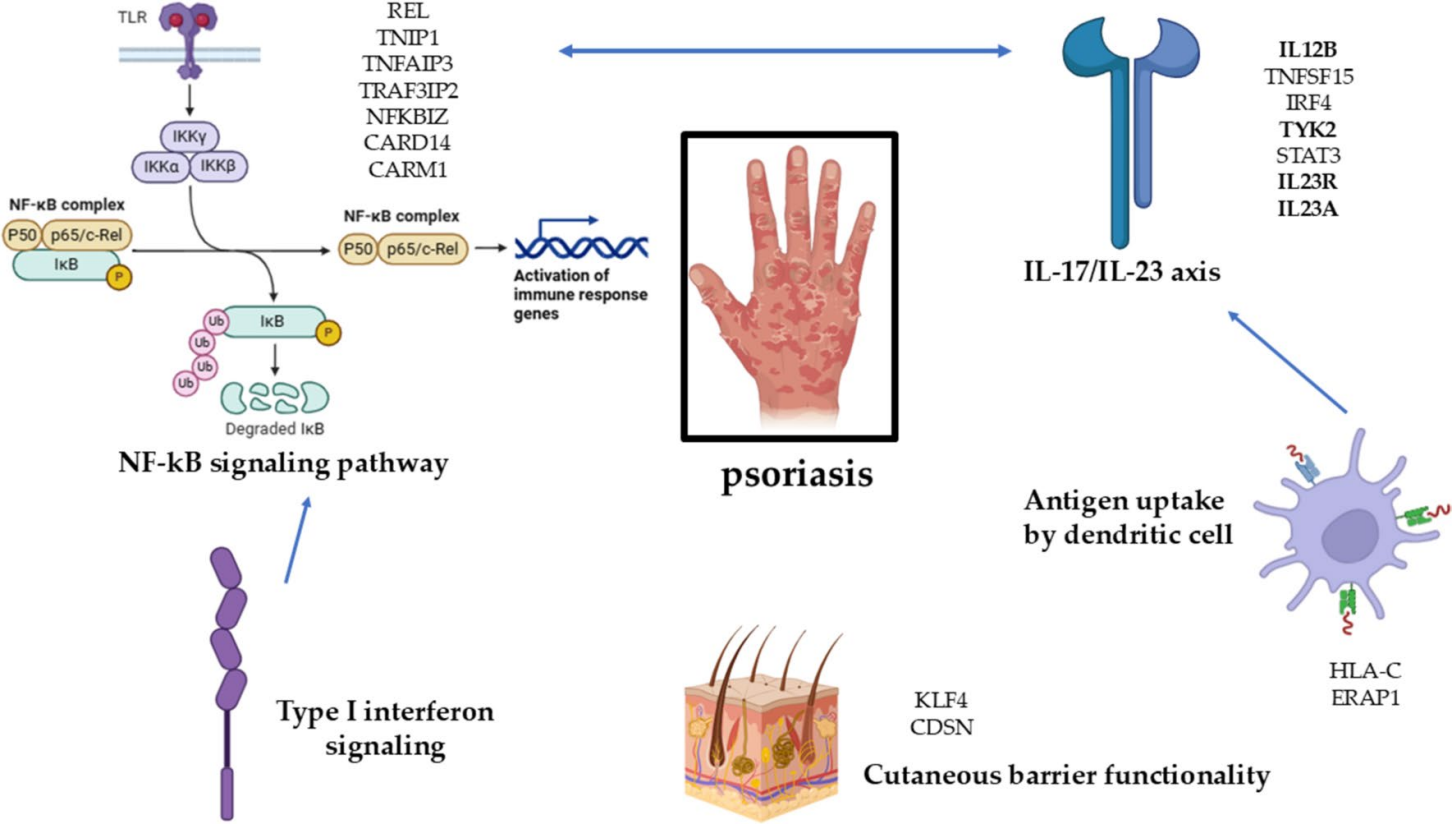
Interrelationships in biological pathways and genetic implications in the pathogenesis of psoriasis. The bolded structures (i.e., IL12B, TYK2, IL23R, and IL23A) are proteins currently addressed by psoriasis treatments, while the arrows indicate the interaction between the depicted immunological pathways. *CARD14* caspase recruitment domain family member 14, *ERAP1* endoplasmic reticulum aminopeptidase 1, *GWAS* genome-wide association studies, *HLA-C* human leukocyte antigen C, *IKK* IκB kinase, *IL-17* Interleukin 17, *IL-23* Interleukin 23, *IL12B* Interleukin 12 subunit beta, *IL23A* Interleukin 23 subunit alpha, *IL23R* Interleukin 23 receptor, *IRF4* Interferon Regulatory Factor 4, *NFKBIZ* NF-kB inhibitor zeta, *NF-kB* nuclear factor kappa-light-chain-enhancer of activated B cells, *p50* NF-kB subunit p50, *p65* NF-kB subunit p65/c-Rel, *REL* regulator of nuclear factor kappa B, *STAT3* signal transducer and activator of transcription 3, *TNFAIP3* TNF alpha-induced protein 3, *TNFSF15* tumor necrosis factor superfamily member 15, *TLRs* toll-like receptors, *TYK2* tyrosine kinase 2, *Th17* T helper 17 cells, *TRAF3IP2* TNF receptor-associated factor 3 interacting protein 2

Additionally, impaired antigen presentation mechanisms contribute to psoriasis susceptibility, notably through genes like HLA-C and ERAP1, which influence the presentation of autoantigens to T cells, further triggering the immune system. Collectively, these intertwined biological pathways create the chronic, relapsing nature of psoriasis, characterized by abnormal immune responses and epidermal hyperplasia. Disruptions in genes such as KLF4 (a regulator of keratinocyte differentiation) and CDSN (which encodes corneodesmosin, crucial for skin integrity) impair the skin’s protective barrier, increasing susceptibility to environmental triggers and perpetuating the inflammatory cycle. Together, these interconnected pathways contribute to the characteristic epidermal thickening, scaling, and inflammation seen in psoriasis (Dand et al. 2020; Babaie et al. 2022). Figure 4 depicts the intricate implications of biological pathways in psoriasis’ pathogenesis.

The discovery of genetic variants associated with complex diseases, including those that are common and uncommon, has been facilitated by advancements in genomic technologies, including whole-exome sequencing, imputation techniques, and next-generation sequencing. Exome sequencing was employed by Tang in a study on a Chinese population to analyze nonsynonymous single nucleotide variants. The study identified two low-frequency missense variants in the IL23R and GJB2 genes that increased the risk of psoriasis (Tang et al. 2014). Moreover, Zhou's investigation of DNA methylation in psoriasis emphasized the substantial correlations between skin-specific methylation patterns and nine differentially methylated sites that are associated with the disease. The expression of genes such as CYP2S1, ECE1, EIF2C2, MAN1C1, and DLGAP4 was inversely correlated with DNA methylation levels, which implies that epigenetic mechanisms are involved in the progression of this condition (Zhou et al. 2016).

**Fig. 5:**
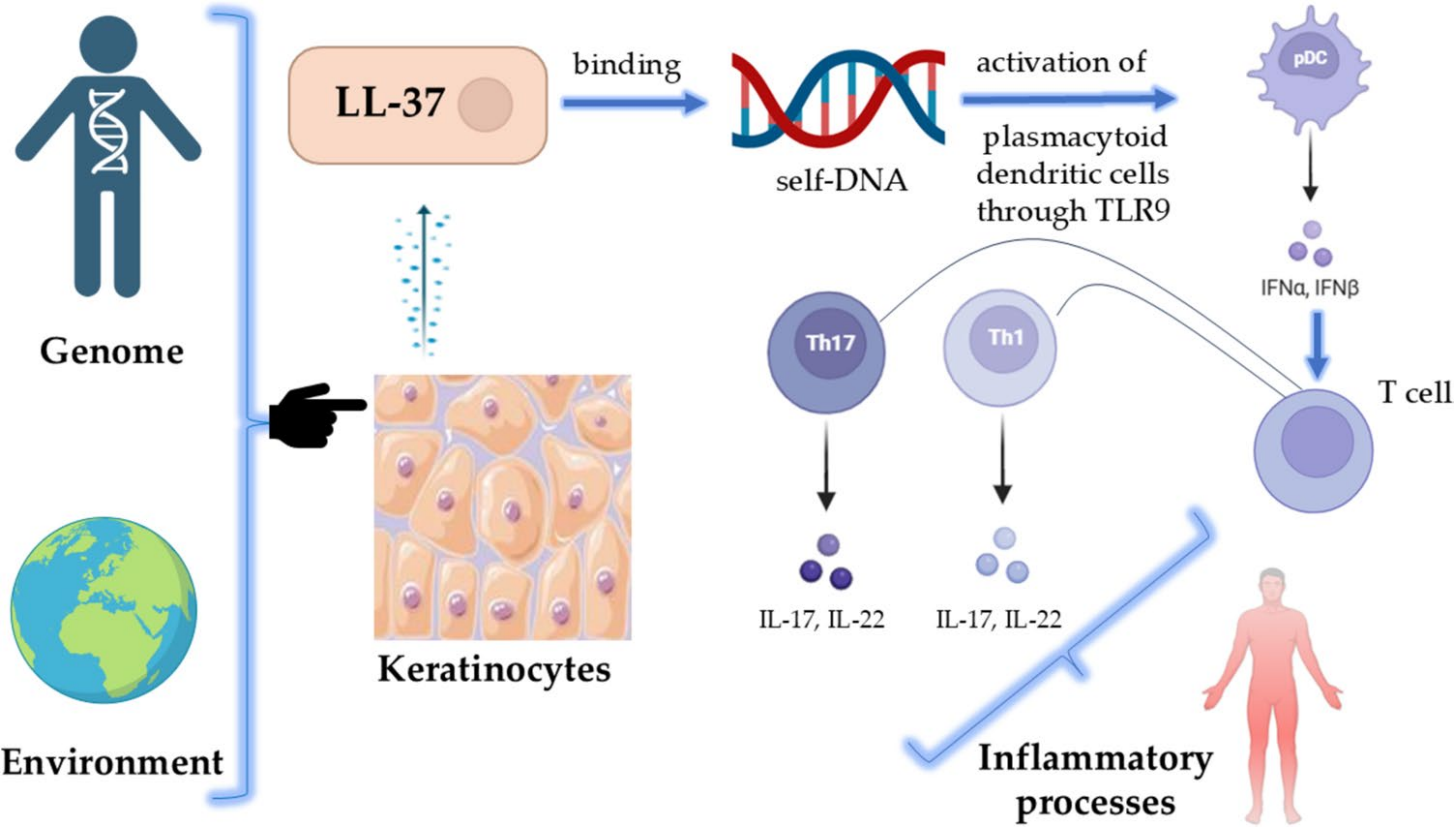
Inflammation mechanisms in psoriasis. *IFN* interferon, *pDC* plasmacytoid dendritic cell, *IL* interleukin, *LL-37* cathelicidin (human antimicrobial peptide)

It is thought that psoriasis may represent a consequence of an intricate interaction between environmental stimuli and genetic characteristics, according to a widely accepted paradigm (Reich 2012; Vičić et al. 2021). Another hypothesis is that keratinocytes may release the antimicrobial peptide LL-37 in response to stressors. This peptide can bind to self-DNA and activate plasmacytoid dendritic cells (pDCs) through TLR9 (Pahar et al. 2020). Activated pDCs release interferon alpha (IFN-α), which stimulates dermal dendritic cells and directs them to lymph nodes, where they stimulate the formation of Th1, Tc, and Th17 from T cell differentiation. Subsequently, once returned to the epidermis, these cells release inflammatory cytokines that facilitate the progression of the disease (Fig. 5) (Ye et al. 2020).

### Treatment choice

The current therapeutic approaches for psoriasis are designed to enhance the standard of life for patients by alleviating skin, joint, and nail lesions. Despite the absence of a definitive curative approach, there are numerous treatment options available. Topical therapies, including corticosteroids, calcineurin inhibitors, and vitamin D3 analogues, as well as non-medicated treatments like moisturizers, coal tar, salicylic acid, and anthralin, are typically employed to manage mild instances which include under 10 percent of the body surface area. Systemic therapies are reserved for severe diseases, and the selection of treatment is determined by patient-specific factors and comorbidities (Zhu et al. 2022).

Corticosteroids operate at the cellular level through both genomic and nongenomic pathways. They activate the glucocorticoid receptor genomically, resulting in its homodimerization and subsequent binding to glucocorticoid-responsive elements. This process facilitates the transcription of anti-inflammatory genes, such as tyrosine aminotransferase, phosphoenolpyruvate carboxy-kinase, and IL-10 (Witchel and DeFranco 2006). Conversely, the nongenomic pathway suppresses the expression of genes that promote inflammation. The most effective topical treatments were potent corticosteroids, which considerably outperformed placebos, according to a comprehensive analysis of trials from 1966 to 1999 by Mason et al. (2002).

The incidence of adverse events was comparatively low for corticosteroids, with a range of 3.2–23%. Fluticasone propionate was at the lower end of this spectrum (Bruner et al. 2003). Corticosteroids classified as Class I, such as halobetasol and clobetasol, are widely recognized as effective treatments for plaque psoriasis. They are typically administered twice daily for 2 weeks, followed by an intermittent regimen to maintain remission. Efficacy has also been demonstrated by mid-potency corticosteroids, such as fluticasone propionate (Lee and Koo 2009).

Predominantly, analogues of vitamin D3 are relied upon for managing the symptoms of mild and moderate forms of psoriasis. These compounds regulate genes that are responsible for cellular proliferation, differentiation, and inflammation by adhering to nuclear vitamin D3 receptors. Calcipotriol, tacalcitol, maxacalcitol, and calcitriol are minimally absorbed systemically and have few adverse effects, with local irritation being the most prevalent (O’Neill and Feldman 2010).

Side effects are minimized while the effectiveness of the treatment is enhanced through the use of combination therapy with corticosteroids (Menter et al. 2009). A fixed combination of betamethasone dipropionate and calcipotriene has demonstrated optimal safety and efficacy for plaque psoriasis in clinical trials (Menter et al. 2013).

Agents like tacrolimus and pimecrolimus, which inhibit calcineurin, are classified as topical treatments and have been identified as effective off-label therapies for sensitive skin regions of the body. These drugs obstruct the stimulation of T-lymphocytes and the synthesis of pro-inflammatory cytokines. Furthermore, moisturizers are crucial in the support of skin health, as they alleviate symptoms of dry, irritated, and cracked skin, improve overall skin health, and increase the efficacy of other topical agents (Amiri et al. 2023).

Since the 1960s, methotrexate (MTX), a systemic agent, has been a primary treatment for severe psoriasis, particularly in conditions such as acute generalized pustular psoriasis and psoriatic arthritis. Although the risk of myelosuppression is a significant concern (Ohbayashi et al. 2010) and leukopenia and thrombocytopenia may manifest at any phase of treatment (Danczak-Pazdrowska 2012), it still has a favorable safety profile (Dogra and Mahajan 2013). The initial dose is typically 7.5 mg per week, with adjustments made in accordance with the patient's response. Furthermore, MTX continues to be a cost-effective treatment of extensive chronic plaque psoriasis (Menting et al. 2015).

It is essential to accurately assess patient eligibility prior to initiating therapy, and all contraindications must be excluded. When abnormalities in laboratory test results are detected, it is frequently adequate to decrease the dosage rather than entirely cease the medication. The medication is intended for the management of all variants of psoriasis, as well as psoriatic arthritis. MTX therapy for psoriasis patients may be administered on an outpatient basis, provided that all dosage and safety guidelines are adhered to. A significant element influencing the efficacy and tolerance of MTX is the method of delivery (Czarnecka-Operacz and Sadowska-Przytocka 2014).

Cyclosporine, a neutral, lipophilic, cyclic undecapeptide, suppresses and reduces inflammation, being among most efficacious systemic medications for psoriasis management (Colombo et al. 2013).

The therapeutic management of psoriasis often necessitates an individualized approach, where both combination and sequential therapies are employed over time to enhance efficacy, ensure safety, and address specific clinical requirements. About 25% of those diagnosed with psoriasis suffer from moderate to severe manifestations of the disease, with the majority necessitating systemic treatments or phototherapy for symptom management (Stern et al. 2004).

The treatment landscape for psoriasis has dramatically shifted with the introduction of biologic agents. These drugs, which specifically target elements of the immune system, represent a significant advancement compared to traditional immunosuppressants. Although generally well tolerated, biologics are proving effective across a range of immune-mediated disorders, with psoriasis being a prime example (Sivamani et al. 2010). The therapeutic management landscape for psoriasis has significantly expanded, with over 10 biologic therapies now authorized by the U.S. Food & Drug Administration (FDA). This encompasses IL-12/23 inhibitors such as ustekinumab, IL-23 inhibitors including tildrakizumab, guselkumab, mirikizumab and risankizumab, IL-17 inhibitors like brodalumab, bimekizumab, ixekizumab and secukinumab, in addition to TNF-alpha inhibitors such as infliximab, etanercept, certolizumab pegol, and adalimumab. As new medicines emerge, the array of therapeutic possibilities expands. (Brownstone et al. 2021; Hawkes et al. 2024). The most recent approvals for use have received from the FDA the following biologic molecules: deucravacitinib (2022) to target moderate to severe plaque psoriasis, spesolimab-sbzo (2022) to address generalized pustular psoriasis flares, tapinarof (2022) to address plaque psoriasis (FDA Novel Drug Therapy Approvals for 2022 2024), bimekizumab (2023) to manage moderate and severe plaque psoriasis in patients qualifying for systemic treatments or phototherapy. (FDA Novel Drug Therapy Approvals for 2023 2024).

Specifically, risankizumab has attained the greatest Psoriasis Area and Severity Index (PASI) scores in both short-term and long-term evaluations. Among all biologics, IL-23 inhibitors have the lowest frequency of side effects and possess the most favorable long-term safety as well as effectiveness characteristics in comparison to TNF-α and IL-17 inhibitors (Wride et al. 2024).

Despite their effectiveness, biologic agents do carry some risks. Patients using TNF-α inhibitors have reported higher incidences of upper respiratory infections, such as pharyngitis, rhinitis, and sinusitis. Moreover, tuberculosis cases have emerged in clinical trials involving these drugs. Long-term safety data on IL-12/23 inhibitors shows that migraines, stiffness in the joints, and infections of the upper respiratory tract are the most common side effects. Similarly, individuals on IL-17 inhibitors face a heightened risk of *Candida* infections, and these medications may potentially worsen or trigger inflammatory bowel disease (Shear et al. 2021).

Recent research has focused on creating novel oral medicines that provide improved efficacy and safety in conjunction with the progress of biologics. Deucravacitinib represents a notable advancement as the inaugural allosteric TYK2 inhibitor sanctioned for adults with moderate-to-severe psoriasis. Furthermore, recent discoveries concerning the biology of psoriasis have catalyzed the development of topical therapies targeting intracellular signaling pathways, such as JAK-STAT, PDE-4 and AhR. Tapinarof, an AhR modulator, and roflumilast, a PDE-4 inhibitor, have both been approved by the FDA for the treatment of plaque psoriasis, exhibiting promising safety and effectiveness profiles. This study examines recent advancements in oral and topical medicines, namely those currently under investigation or recently launched for the treatment of psoriasis (Carmona-Rocha et al. 2024).

Table 1 summarizes few relevant data related to the therapeutic management of psoriasis in different settings formulated and recommended by the National Psoriasis Foundation and the American Academy of Dermatology. These guidelines offer evidence-based strategies tailored to the severity of psoriasis, ensuring comprehensive and effective treatment options for patients.

**Table 1:** Recommendations validated by regulatory bodies targeting topical, conventional systemic, biological and alternative medicine agents for use in psoriasis

Topical agents (Elmets et al. 2021)		
Substance/class	Recommendation	Level of evidence
Topical corticosteroids	For managing plaque psoriasis that does not affect intertriginous regions, topical corticosteroids categorized as classes 1, 2, and 3–5 are suggested for a maximum duration of four weeks	A
	In treating scalp psoriasis, topical corticosteroids classified from classes 1 to 7 are advised for at least four weeks as part of both initial and ongoing therapeutic regimens	A
	When administered under strict medical supervision, extending the use of topical corticosteroids beyond 12 weeks can be acceptable	C
Calcineurin inhibitors	Considering the off-label application of 0.1% tacrolimus for psoriasis on the face and inverse psoriasis for up to eight weeks may provide an effective treatment option	B
	For individuals with inverse psoriasis, prolonged off-label use of pimecrolimus or tacrolimus could be considered	C
	Employing tacrolimus in combination with 6% salicylic acid over a period of 12 weeks may represent an effective treatment method for plaque psoriasis	B
Vitamin D analogues	Patients with mild to moderate psoriasis should apply topical vitamin D analogues like maxacalcitol, calcitriol, calcipotriene, and tacalcitol for 52 weeks	A
	Calcipotriene foam and gel should be used for 4–12 weeks for a mild or moderate form of scalp psoriasis	A
	Combination treatments utilizing calcipotriol alongside corticosteroids are recommended for effective psoriasis management	A
Tazarotene	Patients with mild to moderate psoriasis can benefit from topical tazarotene	B
	For mild to moderate psoriasis, mid- to high-potency topical corticosteroids with tazarotene for 8–16 weeks are more efficacious than tazarotene alone	A
	Combining topical corticosteroids with tazarotene shortens treatment durations and extends remission	A
Moisturizers	Using emollients alongside topical corticosteroids for 4–8 weeks can alleviate itching and flaking, reduce total body surface area involvement, and lessen the chances of psoriasis recurrence after corticosteroid treatment ends	B
Salicylic acid	Mild to moderate psoriasis can be treated with topical salicylic acid for 8–16 weeks	B
	Salicylic acid and topical corticosteroids can treat moderate to severe psoriasis involving up to 20% of the body	B
Anthralin (dithranol)	Anthralin therapy for 8–12 weeks is advised for mild to moderate psoriasis, with a daily contact period of 2 h to reduce side effects	B
	Systemic nonbiologic therapies (Menter et al. 2020)	
Methotrexate	Methotrexate is often prescribed for moderate to severe psoriasis in adults	A
	Compared to adalimumab and infliximab, methotrexate has shown less effectiveness in managing cutaneous psoriasis	A
	Administration of methotrexate can occur either orally or via subcutaneous injections, providing flexibility in treatment delivery	A
	To mitigate gastrointestinal and liver-related adverse effects, the supplementation of folic acid or folinic acid is advised; however, excessive dosages might diminish the therapeutic impact of methotrexate	A
Apremilast	Apremilast is beneficial for moderate to severe psoriasis	A
Cyclosporine	For those suffering from severe and resistant forms of psoriasis, cyclosporine is often recommended	A
	In cases of erythrodermic, palmoplantar, or generalized pustular psoriasis, cyclosporine can also be applied as part of the treatment plan	B
Acitretin	When it comes to plaque psoriasis, acitretin can be recommended as a standalone treatment	B
	Acitretin is also indicated for the management of pustular, palmoplantar, and erythrodermic types of psoriasis, making it a versatile option in treatment regimens	B
	Biologics (Menter et al. 2019)	
Etanercept	Etanercept is an effective solitary treatment for moderate to severe plaque psoriasis	A
	Etanercept and high-potency corticosteroids improve moderate to severe plaque psoriasis treatment	A
	For moderate to severe plaque psoriasis, infliximab is recommended alone	B
Infliximab	As a singular therapy for moderate to severe plaque psoriasis, infliximab is advised	A
	Adults with severe psoriatic arthritis and plaque psoriasis may be administered infliximab to prevent joint destruction detected in imaging investigations	A
Adalimumab	For moderate to severe plaque psoriasis, adalimumab is recommended alone	A
	Adalimumab is also suggested for palmoplantar psoriasis, a moderate to severe plaque psoriasis of the palms and soles	A
Ustekinumab	Ustekinumab is an option for moderate to severe plaque psoriasis in adults	A
	Moreover, ustekinumab can be utilized for adults with plaque-type palmoplantar psoriasis, adding to its therapeutic versatility	B
	When combined with apremilast, ustekinumab can further enhance treatment efficacy for individuals with moderate to severe plaque psoriasis	C
Secukinumab	Adults with moderate to severe plaque psoriasis can treat themselves with secukinumab	A
	Additionally, this medication is indicated for treating moderate to severe psoriasis that affects the scalp, head, and neck regions	B
	For adults dealing with erythrodermic psoriasis, secukinumab is a viable standalone therapy	C
Ixekizumab	For moderate to severe plaque psoriasis, ixekizumab is suggested alone	A
	People with extensive pustular psoriasis may also be candidates for ixekizumab treatment	B
Brodalumab	Brodalumab is recommended as a standalone treatment for moderate to severe plaque psoriasis in adults	A
	Brodalumab could also potentially serve as a monotherapy for generalized pustular psoriasis in adults	B
Guselkumab	Guselkumab is suggested for moderate to severe plaque psoriasis alone	A
	Additionally, guselkumab is effective for addressing psoriasis affecting nails, palms, and the scalp in adults	A
Tildrakizumab	For people with moderate to severe plaque psoriasis, tildrakizumab is suggested alone	A
	Alternative medicine (Elmets et al. 2021)	
Traditional Chinese medicine	Herbal remedies such as, *Indigo naturalis*, *Camptotheca* spp. and *Mahonia aquifolium* have demonstrated anti-inflammatory properties in comparison to a placebo	–
Aloe vera	Topical aloe vera may be useful in mild psoriasis for those without sensitivities to the substance	–
St John’s wort	For individuals with mild psoriasis, the topical application of St. John’s wort may offer some benefits; however, its formulation lacks sufficient standardization for official recommendation	–
Fish/omega-3 oil	The anti-inflammatory characteristics of fish oil, achieved through eicosanoid inhibition, have demonstrated efficacy in psoriasis, especially by oral supplementation	–
Vitamin D supplementation	Although topical vitamin D derivatives prove effective in the treatment of psoriasis, it is not recommended to use oral vitamin D supplements for this specific skin condition	–
Curcumin	Although research is limited, oral curcumin may function as an additional treatment for psoriasis	–
Zinc	The administration of oral zinc supplements has not shown a significant impact on the severity of psoriasis and is therefore not recommended	–

Traditional Chinese medicine (TCM) is among the oldest and most widely utilized supplemental medical practices globally, significantly impacting Asian healthcare practices (Bungau and Popa 2015; Gaur 2024). Nonetheless, substantial empirical data exists that scientific methods cannot quantify, and the absence of standardization has hindered its acceptance by regulatory agencies in numerous nations within mainstream conventional medicine. It is essential to enhance traditional research procedures and methodologies for TCM research to enable its integration, thereby offering the advantages of both paradigms for superior healthcare quality (Chan et al. 2015).

Advancements in biological, chemical, and computational technology necessitate multidisciplinary ways to examine evidence-based elements of TCM treatment. Investigating the correlation between quality control in the production of TCM products, contemporary systems biology, and experiential TCM concepts is essential for understanding the comprehensive framework of TCM in its integration into conventional medicine. Only well-organized and controlled clinical trials can address safety, effectiveness, and standardization concerns, especially with traditional medicines and herbal therapies. Such trials provide strong evidence for clinical practice and regulatory choices, ensuring therapeutic claims are supported by data (Traditional Chinese Medicine Needs Proper Scrutiny 2024).

### Overview of bioactive compounds in plants and food products

For decades, bioactive compounds found in natural extracts have been integral to the pharmaceutical industry, with dermo cosmetics emerging as a key area for their application. The significant advantages of these natural bioactive ingredients in maintaining skin health have led to the creation of a unique category of products known as biocosmetics, or cosmeceuticals. These products occupy an intermediate space between pharmaceuticals and cosmetics, containing biologically active components that closely resemble dermatological treatments in their topical applications (Soto et al. 2018).

Natural compounds, characterized by their small molecular size, can effectively penetrate the skin, offering superior biocompatibility and a facilitated metabolism. They are typically sourced from wild flora or plant residues, supporting sustainable, low-cost production and aligning with zero-waste principles. These compounds are generally well-tolerated and pose fewer long-term safety or environmental risks. However, they often suffer from instability, reduced shelf life, and variable quality, which can be influenced by growth, harvest, and extraction conditions. Additionally, large-scale production is challenging, and natural compounds may occasionally elicit allergic reactions, particularly in individuals with sensitive skin. On the other hand, synthetic compounds are engineered for enhanced stability and longer shelf life, facilitating large-scale, cost-effective production. They can be tailored for specific therapeutic properties and are often more efficacious in targeting particular dermatological conditions. Despite these advantages, synthetic compounds tend to exhibit lower biocompatibility, with a higher potential for adverse reactions, and concerns exist regarding their long-term safety and environmental impact. Furthermore, regulatory oversight for synthetic compounds is limited, contributing to increased consumer demand for safer, natural alternatives in dermatological and pharmaceutical products (Turcov et al. 2023).

When it comes to managing chronic conditions like psoriasis, conventional therapies have been useful in alleviating symptoms, but no treatment has yet provided a complete cure. Additionally, many of the existing treatments come with side effects such as skin atrophy, organ toxicity, immunosuppression, infections, and, in some cases, an increased risk of carcinogenesis, which limits their long-term use. As a result, there is an urgent need to develop safer, more effective, and potentially more affordable treatments for psoriasis.

Studies suggest that herbal remedies and naturally derived bioactive substances, due to their immune-modulation and antioxidant characteristics, may influence the cellular responses associated with psoriasis. A multitude of research evaluations corroborate the efficacy of herbal treatments in the management of psoriasis, highlighting the advantageous benefits of phytochemicals in treating this autoimmune disorder (Aghmiuni et al. 2017).

For psoriasis patients, appropriate food control is essential and must be combined with pharmacological therapy. Patients are encouraged to diminish their consumption of saturated fats and substitute them with polyunsaturated fatty acids (PUFAs), especially omega-3s, recognized for their anti-inflammatory effects. It is advisable to incorporate beneficial components into the diet, including selenium, flavonoids, carotenoids, and vitamins A, E, and C, while also providing enough vitamin D intake. Additionally, diverse alternative dietary approaches, including vegetarian, gluten-free, and Mediterranean diets, have had positive effects on psoriasis management. Dietary strategies must augment pharmacological treatments to improve therapeutic results (Garbicz et al. 2021).

Patients are also encouraged to engage in regular physical activity, avoid alcohol, and consume omega-3-rich fish, fruits, and vegetables. The use of prebiotics and probiotics may offer additional benefits, and in some cases, vitamin D supplementation and a gluten-free diet may be advantageous. Nonetheless, lifestyle and dietary changes should always be viewed as adjunctive measures rather than replacements for conventional treatments (Musumeci et al. 2022).

Chronic inflammation and oxidative stress are major contributors to many non-communicable diseases, including psoriasis. Emerging evidence suggests that certain dietary nutrients can trigger immune responses that lead to the overproduction of pro-inflammatory cytokines. Fatty acids are vital macronutrients that profoundly affect immunomodulation, particularly omega-3 polyunsaturated fatty acids, which are highly beneficial. Conversely, carotenoids and polyphenols function as effective antioxidants that may mitigate oxidative damage. The correlation between psoriasis and obesity is significant, as extra weight can exacerbate clinical manifestations. Thus, measures emphasizing weight reduction and dietary alterations—such as implementing a gluten-free or Mediterranean diet—coupled with suitable supplements, may augment the management of psoriasis and promote patient treatment responses (Katsimbri et al. 2021).

Low-calorie diets, in special, have been shown to enhance the effectiveness of systemic treatments for psoriasis. A medical study demonstrated that obese patients receiving cyclosporine experienced significantly higher response rates when following a low-calorie diet, with 66.7% of patients achieving a PASI75 response, compared to only 29% of patients who were not on a modified diet (*p* < 0.001) (Gisondi et al. 2008).

Improvements in psoriasis symptoms have been associated with adherence to a Mediterranean diet. Research conducted by Barrea et al. indicated that individuals with psoriasis were significantly less likely to follow this dietary pattern (4.8%) compared to healthy controls (30.6%). Reduced consumption of tree nuts, fruits, fish/seafood, and extra virgin olive oil was also reported by the psoriasis group. Notably, a lower intake of fish together with extra virgin olive oil was independently connected to reduced PASI scores, highlighting the potential advantages of a Mediterranean diet for inflammatory diseases (Barrea et al. 2015).

The major implications of diets in inflammatory autoimmune pathologies such as psoriasis are highlighted by their inclusion in clinical studies, which also suggest the importance of further studies in this research direction. In this context, a highlight of clinical trials using the ClinicalTrials.gov database (ClinicalTrials.gov 2024) was pursued, using the search terms 'psoriasis' in the condition/disease box and 'diet' in the 'other terms' section. The database returned 31 studies as results of the search, of which 13 are included in Table 2, the remainder being excluded at the screening stage due to lack of relevance to the addressed topic.

**Table 2:** Clinical trials assessing the efficacy of dietary interventions for psoriasis management

Database ID	Official title	Description	Study type
NCT06399432	Mediterranean diet vs no dietary intervention for improving signs and symptoms of psoriasis in patients treated with anti-IL-17 or anti-IL-23 inhibitors	This investigation aims to assess how the Mediterranean diet influences the effectiveness of anti-IL-17 or anti-IL-23 therapy in patients suffering from psoriasis, especially when compared to a lack of dietary intervention	Int
NCT03531528	A single-arm trial to evaluate the efficacy of an aggressive weight loss program with a ketogenic induction phase for the treatment of chronic plaque psoriasis	Additionally, the research explores whether implementing a rigorous weight loss program that includes a ketogenic phase can enhance treatment results for those dealing with chronic plaque psoriasis alongside obesity	Int
NCT01439425	Weight reduction alone may not be sufficient to maintain disease remission in obese patients with psoriasis: a randomized, investigator-blinded study	The study investigates the relationship between body weight and psoriasis severity using a questionnaire for 200 patients and compares a hypo-caloric diet to a free diet in obese psoriasis patients in remission over 24 weeks	Int
NCT05820698	A feasibility pilot study examining the effect of a Mediterranean style diet and time-restricted eating on individuals with mild-moderate psoriasis	The METRED-P study assesses the feasibility of a Mediterranean diet and/or time-restricted eating in mild to moderate psoriasis over 12 weeks	Int
NCT06574178	Use of total-body PET to quantify systemic and cutaneous inflammation in psoriasis patients before and after intervention with a nutritionally balanced diet	The study aims to assess whether a 6-week dietary intervention following nutrition guidelines can reduce measurable inflammation in psoriasis patients, using the advanced EXPLORER PET scanner technology	Int
NCT05118425	A real-world study of the dietary perceptions and practices in Chinese psoriasis patients	The study conducts a questionnaire-based survey to assess the dietary perceptions and behaviors of Chinese psoriasis patients across 25 medical institutions in China	Obs
NCT05448352	The APPLE study; a cross-sectional study exploring diet, life-style patterns and psoriasis severity	The goal of the study was to explore the relationship between specific dietary practices and lifestyle factors with the severity of psoriasis, utilizing an online survey alongside a four-day dietary log	Obs
NCT05892640	Effects of low salt dietary intake on Th17-mediated inflammation and vascular reactivity in patients with psoriasis	The study investigates the impact of a 2-week low-salt dietary approach on microvascular vasodilation and Th17-mediated inflammation in patients with psoriasis vulgaris	Int
NCT06164860	The effect of ketogenic diet versus Mediterranean diet on clinical and biochemical markers of inflammation and in patients with obesity and psoriatic arthritis	The study compares the effectiveness of the Mediterranean diet and the isocaloric ketogenic diet on inflammation markers in patients with obesity, psoriasis, and psoriatic arthritis over two 8-week periods	Int
NCT01714284	Efficacy study of dietary intervention and weight loss in improving psoriasis	Research is being conducted through a multicenter randomized controlled trial to determine the effectiveness of a structured weight reduction approach versus basic counseling in improving the management of psoriasis	Int
NCT03142503	Effectiveness of a dietary intervention program on disease activity, metabolism and oxidative stress in patients with psoriatic arthritis and psoriasis activity: a clinical, randomized, placebo-controlled trial	Focused on individuals with psoriasis and psoriatic arthritis, this study aims to evaluate the impact of a nutritional intervention program on disease activity, metabolic profiles, and oxidative stress	Int
NCT06257641	Impact of the Mediterranean diet on patients with psoriasis: a randomized clinical trial	In a separate effort, the MEDIPSO trial is designed to explore the benefits of a 16-week high-intensity Mediterranean diet on skin health in participants with mild to moderate psoriasis, comparing the results with those of a control group adhering to standard low-fat dietary guidelines	Int
NCT05590247	Role of intermittent fasting on disease severity and quality of life in psoriasis and psoriatic arthritis	The study evaluates intermittent fasting's effectiveness in improving psoriasis and psoriatic arthritis over 24 weeks, comparing an intermittent fasting group to a standard diet group	Int

*Int* interventional, *Obs* observational

## Specific bioactive compounds targeting psoriasis

### Polyphenols

In combating free radical damage to the skin, plant-derived polyphenols have emerged as vital components. These polyphenolic compounds exhibit a diverse range of biological activities, owing to their varied chemical structures. They play a pivotal role in enhancing skin protection and aiding regeneration, whilst simultaneously eliminating free radicals via several ways (Poljšak and Dahmane 2012).

Research experiments have been conducted to explore the impact of various natural polyphenols on inflammatory and autoimmune disorders. Among these polyphenols, compounds such as caffeic acid, ferulic acid, chlorogenic acid, pelargonin, and resveratrol have demonstrated the ability to modulate the activation of genes associated with inflammation and cytokine production, thereby affecting immune cell profiles (Wen et al. 2020).

Delphinidin, an anthocyanidin that is present in vegetables and fruits that are highly pigmented., was applied to reconstituted human skin, an approach that resulted in enhanced expression and processing of caspases, proteins critical to the skin's cornification process. Delphinidin also promoted the expression of key epidermal differentiation markers (Afaq et al. 2007).

A study utilizing an animal model was conducted to determine the effects of delphinidin when applied topically on the pathological features of psoriatic lesions in flaky skin mice, specifically examining its impact on inflammation, proliferation, and epidermal differentiation. Starting at five weeks of age and continuing until 14 weeks, the mice received topical treatments of delphinidin at doses of 0.5 mg/cm^2^ and 1 mg/cm^2^, administered five times per week. The results revealed that delphinidin significantly reduced markers associated with psoriasis, diminished the infiltration of inflammatory cells, and lowered the levels of pro-inflammatory cytokines at both mRNA and protein stages. Concurrently, treatment with delphinidin enhanced the expression of proteins such as caspase-14, filaggrin, loricrin, and keratins (specifically keratin-1 and keratin-10). Furthermore, a significant decrease in cell proliferation markers and adjustments in tight junction proteins were noted. These results indicate that delphinidin shows potential as a therapeutic agent for psoriasis and other skin conditions marked by excessive cell proliferation (Pal et al. 2015).

Similarly, baicalin, a flavonoid extracted from *Scutellaria baicalensis*, has been shown to have significant biological activities. Psoriasis, a chronic immune-mediated inflammatory skin condition, still lacks treatments that consistently yield positive responses across all patient groups. A murine model of psoriasis was utilized to demonstrate that baicalin could effectively reduce inflammation in the dermis when induced by the topical application of imiquimod (IMQ). Following the five-day treatment with IMQ, the administration of baicalin over an additional four days led to significant improvements in symptoms such as erythema, scaling, and epidermal thickness. The medication significantly reduced pro-inflammatory cytokines production, including as IL-17A, IL-22, IL-23, and TNF-α. Additionally, γδ T cell infiltration into damaged skin was dramatically reduced. Baicalin also suppressed interleukin-17A production in skin-draining lymph nodes (Hung et al. 2018).

Innovative methods in psoriasis treatment have led to the development of a novel topical drug delivery system that integrates curcumin and tea tree oil inside a bi-phasic emulgel formulation. This system has been designed to optimize the therapeutic effects of these natural compounds. The emulgel formulation exhibited superior spreadability and drug release compared to a standard gel formulation. The in vivo studies revealed that the emulgel accelerated the healing of psoriatic lesions more effectively than the traditional gel, suggesting a synergistic interaction between curcumin and tea tree oil that holds potential as a future psoriasis therapy (Reena et al. 2023).

Further research consolidates the therapeutic potential of curcumin in psoriasis treatment. The research encompassed a thorough meta-analysis involving 26 studies, which comprised 7 clinical trials alongside 19 preclinical investigations, demonstrated that both curcumin monotherapy and combination therapy significantly improved PASI scores compared to control groups. Preclinical data showed that curcumin outperformed controls in reducing psoriatic symptoms, modulating cytokine production, and inhibiting cellular proliferation and inflammatory pathways. These results underscore the potential of curcumin as an effective treatment, either independently or in conjunction with conventional treatments (Zhang et al. 2022).

The anti-psoriatic potential of Amentoflavone (AMF), a biflavonoid, has been a subject of investigation in recent studies. In a mouse model induced by imiquimod (IMQ), treatment with AMF led to significant reductions in skin thickness. Observations included improvements in erythema and scaling, alongside a marked decrease in histological markers of inflammation. The mechanism of action appears to involve the inhibition of pro-inflammatory cytokines, specifically IL-22, IL-17A, TNF-α, and IL-23, as well as a suppression of keratinocyte proliferation. Additionally, AMF treatment resulted in the downregulation of the nuclear factor-kappa B (NF-κB) signaling pathway, which is highly involved in the inflammatory process. These findings suggest that AMF could serve as a viable therapeutic option for psoriasis management (An et al. 2016).

Epigallocatechin gallate (EGCG), another promising compound, has demonstrated significant anti-inflammatory, immunoregulatory, and antioxidant properties in psoriasis treatment. EGCG reduced psoriatic dermatitis severity, promoted the expression of caspase-14, and decreased T cell infiltration and cytokines that promote inflammation regulate immune system reaction, while enhancing antioxidant enzyme activity in the plasma (Zhang et al. 2016).

Quercetin (QC), a flavonoid, was shown to ameliorate psoriatic symptoms in an IMQ-induced mouse model by reducing PASI scores and improving histological markers. QC treatment enhanced antioxidant enzyme activities and reduced oxidative stress markers, with its mechanism likely involving the downregulation of the NF-κB pathway (Chen et al. 2017a).

Taxifolin (TXL) has also been found to exert anti-psoriatic effects by inhibiting abnormal keratinocyte proliferation and modulating Th cell differentiation. Research demonstrated that TXL effectively reduces the activity of pro-inflammatory transcription factors such as RORγt, T-bet, and GATA-3. This action leads to a notable decrease in inflammation observed in a murine model of psoriasis (Yuan et al. 2020).

Kaempferol, another natural flavonol, has been explored for its anti-psoriatic potential. In an IMQ-induced psoriasis mouse model, kaempferol reduced skin lesions, suppressed pro-inflammatory cytokines, and increased regulatory T cell populations. This highlights its potential as a future therapeutic agent for psoriasis (Liu et al. 2019).

Genistein, a soy-derived flavonoid, has also shown promise in psoriasis treatment. In both in vitro and in vivo investigations, a reduction in epidermal thickness was documented, along with a decrease in the expression of inflammatory factors and a suppression of keratinocyte proliferation. The effects of genistein seem to be facilitated by its ability to inhibit the NF-κB and STAT3 signaling pathways, that are vital in regulating inflammation and cell proliferation in psoriatic ski. Taken together, these findings suggest that natural polyphenols and flavonoids hold significant potential in treating psoriasis by targeting key inflammatory pathways, modulating immune responses, and promoting skin barrier function. Further exploration and development of these compounds may lead to novel, effective therapies for this chronic skin condition (Wang et al. 2019).

Flavonoids, a substantial category of bioactive compounds examined for their antioxidant as well as anti-inflammatory effects, have been distinctly categorized in Table 3 according to in vitro and in vivo outcomes conducted on specific animal models.

**Table 3:** Flavonoids evaluated in different experimental models for potential use in psoriasis

Substance	Experimental model	Outcomes	References
In vitro			
Cyanidin	Primary human keratinocytes	The activity of all five LCE3 genes shows a significant increase	Austin et al. (2014)
Luteolin	TNF-triggered human keratinocytes	There is a marked reduction in the generation of proteins such as IL-6, IL-8, and VEGF	Weng et al. (2014)
Delphinidin	Normal human epidermal keratinocytes	Improvements in the cornification process led to elevated protein levels of specific markers associated with this process, notably caspase-14 and keratin	Chamcheu et al. (2013)
	Complete three-dimensional reconstructed human dermis	The induction of differentiation markers, including caspase-14, filaggrin, loricrin, and involucrin, occurs at both the mRNA and protein levels	Chamcheu et al. (2015)
Baicalein	HaCaT keratinocytes	Levels of keratin 1 and keratin 10 (K1/K10) rise, accompanied by increased phosphorylation of key proteins like ERK, AKT, and p38 MAPK	Huang et al. (2016)
Genistein	HaCaT cells	The activation of reactive oxygen species is inhibited, resulting in decreased RNA and IL-8, IL-20, and CCL2	Smolińska et al. (2018)
Topical administration			
Epigallocatechin-3-gallate	IMQ-induced BALB/c mice	The use of topical treatments results in a decrease in epidermal PCNA levels while simultaneously enhancing the expression of caspase-14	Zhang et al. (2016)
Naringenin	IMQ-induced BALB/c/IMQ-stimulated keratinocytes	The overproduction of IL-6 is brought back to normal baseline levels	Alalaiwe et al. (2020)
Glabridin	IMQ-induced BALB/c mice/HaCaT cells	Several cytokines, such as IL-1β, CCL2, IL-23, TNF-α, IL-6, IL-22, and IL-17A, exhibit suppressed expression	Li et al. (2018)
Proanthocyanidin	IMQ-induced BALB/c mice	There is a notable decline in the infiltration of inflammatory cells, coupled with the downregulation of psoriasis-related genes including IL17a, IL22, S100a9, and Krt1, alongside the inhibition of arachidonate 5-lipoxygenase activity	Toda et al. (2020)
Delphinidin	Flaky skin mice (fsn/fsn)	Pathological indicators within psoriasiform lesions are diminished, leading to a decrease in both inflammatory cell presence and the expression of inflammatory cytokines at both mRNA and protein levels	Pal et al. (2015)
Rhododendrin	IMQ-induced C57BL/6 mice	The infiltration of inflammatory mononuclear cells is reduced, along with a decrease in pro-inflammatory mediator expression, while the activation of the TLR-7/NF-κB and mitogen-activated protein kinase pathways is inhibited	Jeon et al. (2017)
Oral administration			
Quercetin	IMQ-induced BALB/c mice	Oral administration results in the downregulation of NF-κB, IKKα, NIK, and RelB, while promoting the expression of TRAF3	Chen et al. (2017a)
Amentoflavone	IMQ-induced BALB/c mice/M5 -treated HaCaT cells	The increased levels of cyclin D1, cyclin E, IL-17A, and IL-22 are suppressed, along with a decrease in NF-κB expression	An et al. (2016)
Hesperidin	IMQ-induced BALB/c mice/LPS-stimulated HaCaT cells	Serum levels of leptin, adiponectin, and resistin are regulated, while the activation of IRS-1/ERK1/2 is inhibited	Li et al. (2019)
Taxifolin	IMQ-induced BALB/c mice/LPS-induced HaCaT cells	Inhibition occurs in the Notch1 and JAK2/STAT3 signaling pathways	Yuan et al. (2020)
Hispidulin	IMQ-induced C57BL/6J mice/Activated keratinocytes	Pathologically elevated immunoglobulin G2a, myeloperoxidase, and TNF-α levels are significantly reduced, alongside diminished Th1 and Th17 cell populations	Kim et al. (2020)
Quercitrin	IMQ-induced C57BL/6 mice	Cytokine expression associated with psoriasis, particularly those involved in the IL-23/Th17 axis, is lowered, leading to a decrease in Th17 cell activity mediated by the JAK/STAT signaling pathway	Chen et al. (2017c)
Astilbin	IMQ-induced BALB/c mice	The heightened levels of circulating CD4 + and CD8 + T cells, as well as inflammatory cytokines are notably improved	Di et al. (2016)

**Fig. 6:**
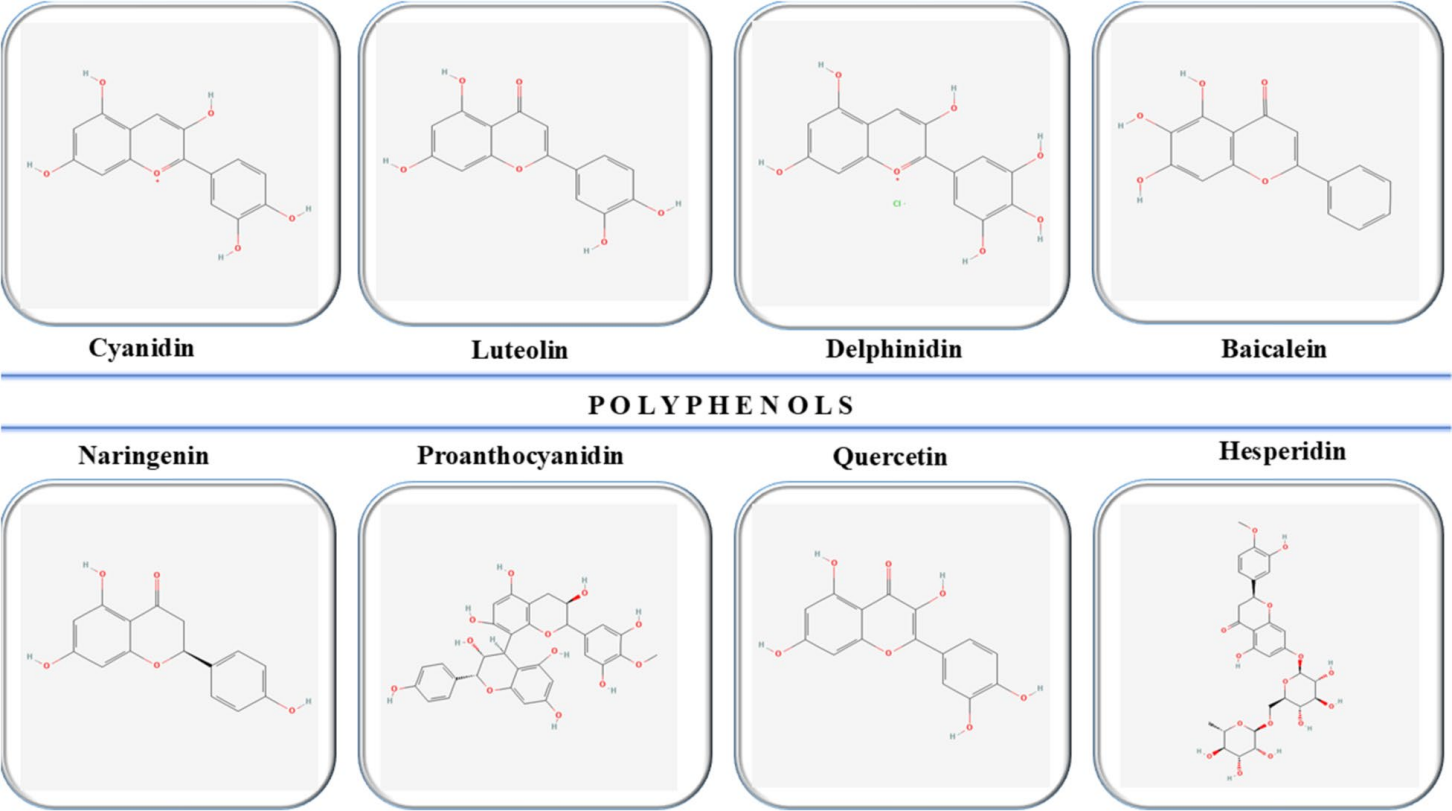
Chemical structures of key natural polyphenols in psoriasis

Figure 6 illustrates the chemical structures of some of the most relevant natural compounds of the polyphenol class (PubChem 2024).

### Terpenoids

The effects of paeoniflorin (PF) on IMQ-induced psoriasis were studied. Four groups of mice treated with IMQ received intragastric 240 mg/kg/day or 120 mg/kg/day PF, 1 mg/kg/day MTX, or saline as a control. Additionally, a vaseline-treated control group of mice with similar weight was used for comparison. The research evaluated various aspects, such as morphological alterations, tissue integrity, keratinocyte proliferation, immune cell infiltration, and mRNA levels of Th1/Th2/Th17/Treg cytokines, along with protein phosphorylation linked to Th17 cell differentiation. Mouse spleen cells were grown under Th17 polarizing conditions and treated with PF at 2, 20, and 200 μg/mL doses. The assessments determined cell survival, Th17 cell differentiation, cytokine production, and RORγt mRNA expression. PF administration led to a significant reduction in keratinocyte overgrowth and immune cell infiltration in IMQ-treated skin, as well as a decrease in Th17 cytokine mRNA levels starting from day four. PF also diminished protein phosphorylation involved in Th17 differentiation. Interestingly, PF, at doses of 2 and 20 μg/mL, reduced IL-17 secretion without affecting spleen cell viability, and it also suppressed Th17-related cytokine mRNA expression and lowered STAT3 phosphorylation in spleen cells. Overall, PF’s anti-psoriatic effects were attributed to its regulation of the Th17 response and cytokine secretion, largely through controlling STAT3 phosphorylation (Zhao et al. 2016).

Another study explored the effects of artesunate (ART), a derivative of artemisinin commonly used for malaria treatment, in the context of psoriasis-like dermatitis induced by IMQ, a TLR7/8 agonist. ART is recognized for its anti-inflammatory and immunomodulatory properties in autoimmune diseases, although its potential therapeutic role in psoriasis had not been previously examined. In this study, it was observed that BALB/c mice treated with ART exhibited a significant reduction in cumulative psoriasis scores, epidermal thickening, and Ki-67 expression when compared to the IMQ psoriatic model group. Additionally, ART treatment mitigated systemic inflammatory responses in the treated mice. Mechanistically, ART was found to reduce the presence of γδ T cells in draining lymph nodes, a change that likely contributed to the improvement of psoriasis-like dermatitis in the mice. These results suggest that ART holds potential as a clinical treatment option for psoriasis, offering a new avenue for therapeutic intervention in this chronic inflammatory skin disorder (Huang et al. 2019).

### Omega-3 fatty acids

A clinical trial examined the efficacy of an omega-3-rich supplement in mild to moderate plaque psoriasis. The trial involved 30 patients, with one group of 15 patients receiving only topical tacalcitol as a control, while the other 15 patients were treated with both tacalcitol and two daily capsules of Oravex^®^. The study measured several key outcomes, including the PASI, Nail Psoriasis Severity Index (NAPSI), and the Dermatological Life Quality Index (DLQI). Both groups exhibited significant and meaningful improvements across all measured parameters from the beginning of the trial to its conclusion. Importantly, the group that was supplemented with Oravex® showed substantially greater improvements compared to the control group. The addition of omega-3 fatty acids to the topical therapy led to a notable reduction in PASI and NAPSI scores, improved DLQI, and further relief from symptoms such as scaling, pruritus, erythema, and scalp lesions in the areas affected by psoriasis (Balbás et al. 2011).

A comprehensive meta-analysis was conducted to assess the effectiveness of omega-3 fatty acids in managing psoriasis. Both fixed-effects and random-effects models were utilized to perform combined and stratified analyses, aiming to calculate the overall effect sizes. From the reviewed literature, 10 studies involving a total of 560 participants were selected for inclusion. The results of the meta-analysis indicated a significant decrease in PASI scores, with a reduction of − 1.58, favoring the participants who received omega-3 PUFA supplementation over those who did not. The random-effects model also indicated a notable reduction in erythema (− 1.66 units) and scaling (weighted mean difference − 0.69), with a significant impact observed particularly in studies employing higher dosages of omega-3 supplementation. These findings support the therapeutic benefit of omega-3 PUFA in alleviating key symptoms of psoriasis such as erythema, itching, and scaling. However, additional well-controlled and randomized studies are required to validate results, particularly those that yielded non-significant or ambiguous outcomes (Clark et al. 2019).

A related study measured the content and composition of 14 serum fatty acids in 85 patients experiencing exacerbated plaque psoriasis and compared them to 32 healthy controls, using gas–liquid chromatography and a flame-ionization detector. The fatty acids (FAs) were categorized based on their biological properties into saturated FAs (SFA), unsaturated FAs (UFA), monounsaturated FAs (MUFA), n-3 polyunsaturated FAs (n-3 PUFA), and n-6 PUFA. The study found significant deviations in FA profiles in psoriatic patients, with and without obesity, when compared to healthy controls. In non-obese patients, a correlation between lower levels of circulating docosahexaenoic acid (DHA) and n-3 PUFA and higher MUFA levels was identified. Additionally, the SFA/UFA ratio was shown to increase with the duration of the disease across all psoriatic patients. These findings suggest that abnormal FA profiles may reflect underlying metabolic imbalances and contribute to the comorbidities observed in psoriasis (Myśliwiec et al. 2017).

A review of 18 randomized controlled trials, including 927 participants, assessed the potential benefits of fish oil and its omega-3 polyunsaturated fatty acids (PUFAs) for treating psoriasis. The findings revealed that when fish oil or omega-3 PUFAs were used as a standalone treatment, there was no statistically significant improvement in PASI scores (*p* = 0.47), lesion size (*p* = 0.34), or itching (*p* = 0.62). However, notable improvements were observed when fish oil or omega-3 PUFAs were combined with standard psoriasis therapies, resulting in a meaningful decrease in PASI scores (mean difference − 3.92) and lesion size (mean difference − 30.00). These results suggest that fish oil or omega-3 PUFAs may be more effective as adjunctive treatments rather than as monotherapies. Furthermore, safety assessments indicated no notable differences between treatment groups. Fish oil supplementation also contributed to mitigating risk factors associated with obesity, cardiovascular disease, and metabolic disorders in psoriasis patients, while regulating several key inflammatory mediators (Chen et al. 2020).

Fish oil and omega-3 PUFAs, when administered alongside conventional therapies, show promising benefits in the treatment of psoriasis and its associated comorbidities conditions such as obesity, cardiovascular complications, and metabolic syndrome. These findings support the potential use of omega-3 PUFAs as an adjunct therapy in psoriasis treatment, though further research is needed to establish their standalone efficacy in psoriatic management.

### Alkaloids

Khasianine, a compound known for its potent anti-inflammatory abilities, was explored for its effectiveness in reducing psoriasis-like skin inflammation in mice. Researchers utilized both an IMQ-induced mouse model of psoriasis and human keratinocyte cultures. Detailed assessments were conducted using immunohistochemical and immunofluorescence techniques to observe pathological changes in psoriatic skin after khasianine administration. In vitro, TNF-α-stimulated HaCaT cells helped examine NF-κB p65's cellular positioning and expression, alongside the levels of IL-17A and IL-33. To better understand how khasianine works at the molecular level, scientists also looked at NF-κB p65's binding affinity to IL-17A and IL-33 promoters. Results revealed that khasianine significantly decreased the infiltration of CD4 + T helper cells and macrophages within the psoriatic lesions in the mice. Further immunohistochemical analysis indicated reduced TNF-α levels, inhibition of NF-κB p65 activation, and lower expression of IL-17A and IL-33 in epidermal keratinocytes. The in vitro experiments showed that khasianine blocked TNF-α-induced NF-κB p65 activation by preventing its attachment to the promoters of IL-17A and IL-33, thus inhibiting NF-κB from moving into the nucleus. Overall, these results highlight the strong anti-inflammatory properties of khasianine, suggesting it could be a valuable topical treatment option for psoriasis (Yang et al. 2022).

An alkaloid-rich fraction, INM-A, was extracted from Qing Dai for creating a natural therapeutic approach for psoriasis. INM-A's chemical composition and anti-psoriatic efficacy were tested both in vivo and in vitro. Furthermore, UV–VIS spectrophotometry and HPLC were used to measure alkaloid content. In vivo, an IMQ-induced psoriatic mouse model was used to evaluate INM-A’s efficacy, while cellular oxidative stress and cytokine responses were measured in vitro to determine its mechanism of action. Containing seven alkaloid compounds, INM-A notably enhanced skin conditions in IMQ-induced mice by lowering IL-17A levels in both psoriatic models and polarized Th17 cells. Additionally, INM-A focused on IL-17A to reduce inflammation and oxidative stress driven by oxidative phosphorylation in human keratinocytes, highlighting its therapeutic promise (Lee et al. 2024).

Cell proliferation in psoriasis involves DNA replication initiation, which is regulated by the assembly of the prereplication complex. CDC6, a critical regulator of the prereplication complex assembly in eukaryotic cells, plays a pivotal role in keratinocyte proliferation, although its involvement in psoriasis was previously unclear. In an in vitro study, CDC6 expression was found to be elevated in psoriatic epidermal cells and inducible by IL-22/STAT3 signaling, a pathway strongly associated with psoriasis pathogenesis. Silencing of CDC6 led to reduced keratinocyte proliferation. Berberine (BBR) was identified as an inhibitor of the CDK4/6-RB-CDC6 signaling axis, reducing keratinocyte proliferation by inhibiting JAK1, JAK2, and TYK2, which subsequently blocked STAT3 activation. BBR effectively inhibited IMQ-induced psoriasis-like skin lesions and reduced CDC6 and phosphorylated STAT3 levels in mice. These results underscore the role of CDC6 in psoriasis and highlight BBR's potential as a therapeutic agent by targeting JAK-STAT3 signaling and CDC6 expression (Sun et al. 2019).

Oxymatrine, a bioactive compound extracted from *Sophora flavescens*, has demonstrated multiple beneficial properties, including inflammation reduction, inhibition of cell proliferation, regulation of the immune response, and prevention of tumor development. A retrospective analysis of Oxymatrine’s effects on psoriasis confirmed a significant reduction in the PASI score compared to controls. Oxymatrine was shown to inhibit human keratinocyte viability, proliferation, and differentiation in vitro, while immunohistochemical analysis revealed suppressed expression of pan-cytokeratin, p63, and keratin 10. In particular, the inhibition of p63 is believed to mediate oxymatrine's anti-proliferative effects on keratinocytes. Notably, oxymatrine did not disrupt basement membrane formation, preserving the normal function of keratinocytes. These outcomes make oxymatrine an effective, affordable, and safe option for treating intractable psoriasis vulgaris (Chen et al. 2017b).

**Fig. 7:**
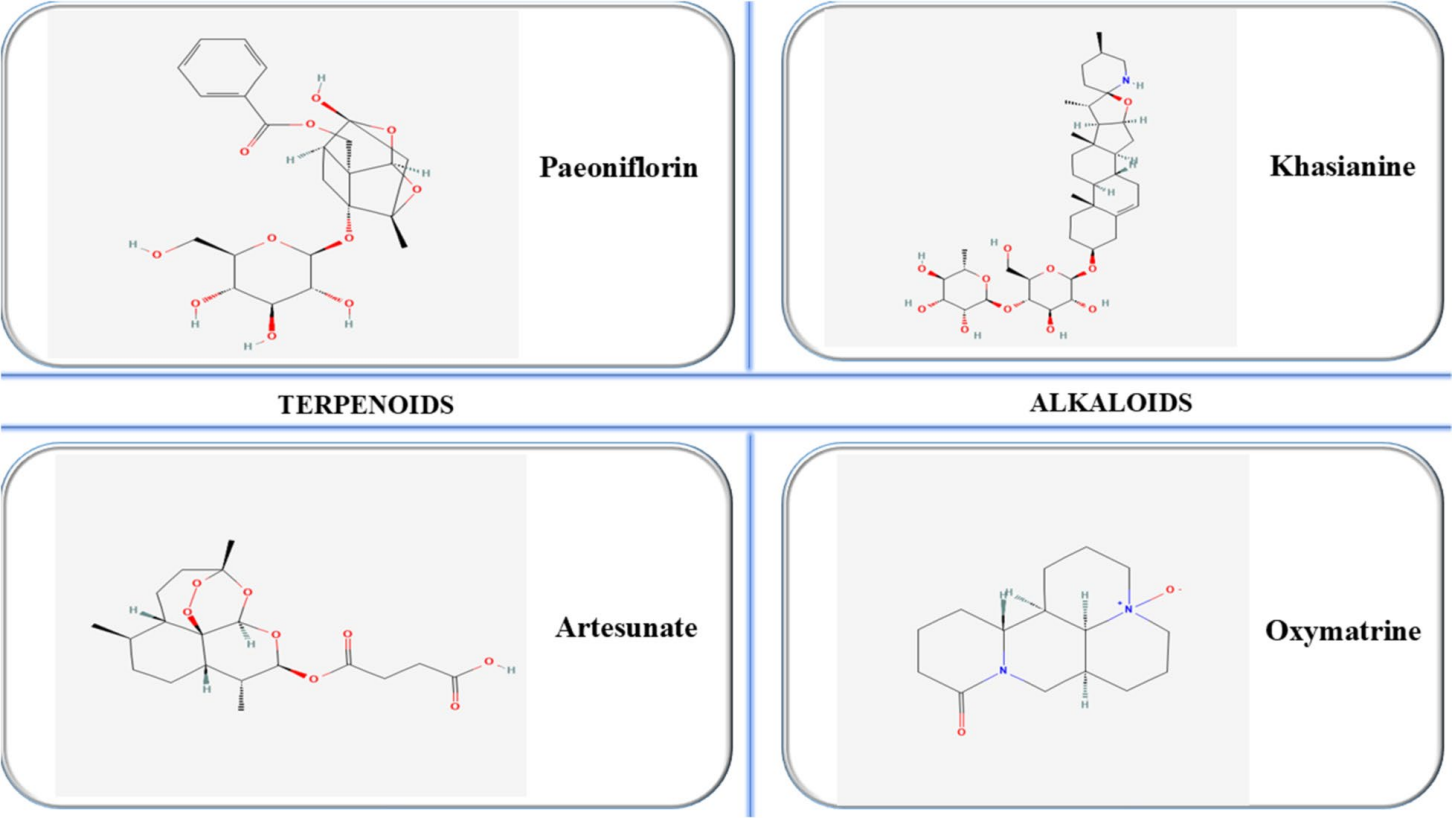
Chemical structures of key terpenoids and alkaloids in psoriasis

Figure 7 illustrates the chemical structures of some of the most relevant natural compounds of the polyphenol class (PubChem 2024).

Table 4 highlights the correlation between bioactive compounds, mechanism of action and observed therapeutic effect in inflammatory contexts.

**Table 4:** Pharmacology of individual bioactive compounds in inflammation

Bioactive compound	Mechanism of action	Therapeutic effects	References
Amentoflavone	Inhibits pro-inflammatory cytokines and keratinocyte proliferation	Reduces skin thickness and inflammation	An et al. (2016)
Baicalin	Reduces inflammation and cytokine production	Alleviates psoriasis symptoms, reduces erythema and scaling	Hung et al. (2018)
Caffeic acid	Modulates inflammation and cytokine production	Reduces inflammatory response	Wen et al. (2020)
Chlorogenic acid	Modulates inflammation and cytokine production	Reduces inflammatory response	Wen et al. (2020)
Curcumin	Modulates cytokine production and inflammatory pathways	Improves PASI scores, reduces psoriatic symptoms	Zhang et al. (2022)
Delphinidin	Enhances caspase expression, promotes epidermal differentiation	Reduces psoriasis markers, inflammation, and promotes skin differentiation	Afaq et al. (2007) and Pal et al. (2015)
Epigallocatechin gallate	Exhibits anti-inflammatory and immunoregulatory properties	Reduces dermatitis severity and enhances antioxidant activity	Zhang et al. (2016)
Ferulic acid	Modulates inflammation and cytokine production	Reduces inflammatory response	Wen et al. (2020)
Genistein	Inhibits NF-κB and STAT3 signaling pathways	Reduces epidermal thickness and inflammatory factors	Wang et al. (2019)
Kaempferol	Reduces pro-inflammatory cytokines and increases regulatory T cells	Reduces skin lesions in psoriasis	Liu et al. (2019)
Quercetin	Reduces oxidative stress and modulates NF-κB pathway	Ameliorates psoriatic symptoms, improves histology	Chen et al. (2017a)
Resveratrol	Modulates inflammation and cytokine production	Reduces inflammatory response	Wen et al. (2020)
Taxifolin	Modulates Th cell differentiation and inhibits pro-inflammatory factors	Decreases inflammation in psoriasis model	Yuan et al. (2020)
Artesunate	Reduces γδ T cells and systemic inflammation	Decreases psoriasis scores, epidermal thickening, and Ki-67 expression	Huang et al. (2019)
Berberine	Inhibits CDK4/6-RB-CDC6 signaling axis and JAK-STAT3 activation	Reduces keratinocyte proliferation and IMQ-induced psoriasis lesions	Sun et al. (2019)
Khasianine	Inhibits NF-κB p65 activation and decreases IL-17A and IL-33 expression	Reduces inflammation and immune cell infiltration in psoriasis lesions	Yang et al. (2022)
Omega-3 fatty acids	Modulates inflammation and immune responses	Improves PASI and NAPSI scores, alleviates symptoms like scaling and pruritus	Balbás et al. (2011) and Clark et al. (2019)
Oxymatrine	Inhibits keratinocyte proliferation and regulates immune response	Reduces PASI score, maintains normal keratinocyte function	Chen et al. (2017b)
Paeoniflorin	Regulates Th17 response and cytokine secretion, affects STAT3 phosphorylation	Decreases keratinocyte overgrowth and immune cell infiltration	Zhao et al. (2016)
INM-A	Reduces IL-17A levels, alleviates inflammation and oxidative stress	Improves skin conditions in IMQ-induced mice	Lee et al. (2024)

*IL* interleukin, *IMQ* imiquimod, *JAK* Janus kinase, *NAPSI* Nail Psoriasis Severity Index, *NF-κB* nuclear factor kappa-light-chain-enhancer of activated B cells, *PASI* Psoriasis Area Severity Index, *STAT3* signal transducer and activator of transcription 3, *Th* T helper, *INM-A* an alkaloid-rich phytopharmaceutical prepared from Qing Dai

### Safety and toxicity issues

Despite the general belief that natural medicinal products are relatively low risk, there are signs of growing awareness of the potential hazards related to this sort of compounds as the use of natural remedies continues to increase. Herbal products carry the risk of potential adverse effects due to several factors, including their intrinsic toxicity. Additionally, issues such as adulteration, incorrect identification of plant species, contamination, and interactions with other natural remedies or synthethic drugs can contribute to harmful outcomes (Jordan et al. 2010).

Significant obstacles may arise when herbal medicinal products are suspected of being linked to adverse events. Natural products frequently incorporate numerous botanicals or other ingredients. In such instances, it may be feasible to attribute causality to the product in its entirety rather than to individual ingredients. It is crucial to identify the plant part utilized, regardless of products that contain only one ingredient (Ekor 2014).

Numerous natural remedies are recognized for their toxicity to humans. The Encyclopaedia of Materia Medica provides extensive documentation, noting that 495 out of 5767 medicinal herbs possess toxic properties. For instance, aristolochic acid, derived from *Aristolochia debilis*, is linked to acute kidney failure, as evidenced by cases involving women who consumed the same weight-loss supplement. Similarly, *Glycyrrhiza* spp., commonly used in TCM for its expectorant properties, contains mineralocorticoids that can result in adverse effects like hypertension and fluid retention. Furthermore, *Panax ginseng*, a widely utilized herb for conditions such as fatigue and poor circulation, has been associated with insomnia and irritability (Zhou et al. 2019).

A cross-sectional survey evaluating over-the-counter natural remedies-related adverse events reported gastrointestinal problems, allergic reactions and dizziness among the most common adverse reactions (Kim et al. 2013). Furthermore, when administered systemically, *Trigonella arabica* species has been observed to exhibit a hypoglycemic effect (Puri et al. 2002). In patients with psoriasis, oral curcumin proved to be well tolerated and harmless. All adverse events were modest and restricted to gastrointestinal discomfort and heat intolerance (Kurd et al. 2008).

The current increase in scientific interest in flavonoids is attributed to their acknowledged antioxidant and estrogenic effects, prompting recommendations for their application as anticarcinogenic and cardioprotective compounds. As a result, the intake of flavonoids as dietary supplements has risen markedly. The potential harmful effects linked to excessive use are frequently disregarded. At high quantities, flavonoids may demonstrate mutagenesis characteristics, function as pro-oxidants producing free radicals, and impede essential enzymes responsible for hormone regulation (Skibola and Smith 2000).

Patients may employ both traditional and mainstream treatment simultaneously, thus heightening the risk of adverse responses stemming from interactions between herbal remedies and medications (Izzo and Ernst 2001). *Salvia miltiorrhiza* has been documented to augment the anticoagulant effects of warfarin (Chan 2001). Additionally, *Ginkgo biloba* has been shown to interact with ibuprofen, potentially leading to fatal intracerebral hemorrhage (Meisel et al. 2003). The ambiguity surrounding the mechanisms of these interactions presents substantial safety risks for persons who self-medicate without appropriate guidance and supervision. In order to prevent the safety concerns that arise from inappropriate use and practice, it is imperative to ensure that the education level is maintained and the regulation targeting natural bioactive compounds is strengthened.

## Conclusions

Bioactive compounds offer significant potential in managing the inflammatory processes associated with psoriasis, an incurable and chronic autoimmune disease. Their immunomodulatory and antioxidant properties provide a promising avenue for reducing inflammation and improving patient outcomes, particularly when used alongside conventional treatments. Although existing therapies have made notable progress, the necessity for safer, more effective alternatives is underscored by the fact that numerous have been linked to adverse consequences. The growing interest in natural compounds, especially in polyphenols, omega-3 fatty acids, and terpenoids, highlights their potential in addressing unmet needs in psoriasis management. However, further research is essential to fully understand their mechanisms, optimize their therapeutic potential, and ensure their safety in long-term use. Continuing studies in this field are crucial to developing novel treatment strategies that can better enhance the quality of life of individuals as well as exert control over the condition.

## Data Availability

Not applicable.
